# Enhancing the Understanding of Breast Vascularity Through Insights From Dynamic Contrast-Enhanced Magnetic Resonance Imaging: A Comprehensive Review

**DOI:** 10.7759/cureus.70226

**Published:** 2024-09-26

**Authors:** Pratiksha Sachani, Rajasbala Dhande, Pratapsingh Parihar, Paschyanti R Kasat, Gautam N Bedi, Utkarsh Pradeep, Prachi Kothari, Smruti A Mapari

**Affiliations:** 1 Radiodiagnosis, Jawaharlal Nehru Medical College, Datta Meghe Institute of Higher Education & Research, Wardha, IND; 2 Medicine, Jawaharlal Nehru Medical College, Datta Meghe Institute of Higher Education & Research, Wardha, IND; 3 Medicine, Lister Hospital, Stevenage, GBR; 4 Obstetrics and Gynecology, Jawaharlal Nehru Medical College, Datta Meghe Institute of Higher Education & Research, Wardha, IND

**Keywords:** breast cancer imaging, breast vascularity, dynamic contrast-enhanced mri (dce-mri), perfusion analysis, treatment response monitoring, tumor angiogenesis

## Abstract

Breast vascularity plays a crucial role in both physiological and pathological processes, particularly in the development and progression of breast cancer. Understanding vascular changes within breast tissue is essential for accurate diagnosis, treatment planning, and monitoring therapeutic response. Dynamic contrast-enhanced magnetic resonance imaging (DCE-MRI) has emerged as a valuable tool for evaluating breast vascularity due to its ability to provide detailed functional and morphological insights. DCE-MRI utilizes contrast agents to highlight blood flow and vessel permeability, making it especially useful in differentiating between benign and malignant lesions. This review explores the significance of DCE-MRI in breast vascularity assessment, highlighting its principles, clinical applications, and role in detecting malignancy through vascular changes. We also examine its utility in monitoring treatment response and quantitative analysis of perfusion metrics such as Ktrans and extracellular-extravascular volume (Ve). While DCE-MRI offers remarkable diagnostic accuracy, challenges remain regarding its cost, accessibility, and potential overlap of enhancement patterns between benign and malignant conditions. The review further discusses emerging technologies and future directions for DCE-MRI, including advanced imaging techniques and machine learning-based quantification. Overall, DCE-MRI stands out as a powerful tool in the comprehensive evaluation of breast vascularity, with significant potential to improve patient outcomes in breast cancer management.

## Introduction and background

Breast vascularity refers to the network of blood vessels that supply the breast tissue, playing a vital role in maintaining its physiological functions. This vasculature undergoes dynamic changes throughout a woman's life, influenced by hormonal fluctuations, pregnancy, and menopause [1]. In pathological conditions, especially breast cancer, the vascular network becomes highly irregular, with abnormal vessel growth and increased permeability, often linked to tumor angiogenesis. Understanding these vascular changes is crucial for diagnosing and managing breast diseases, particularly malignancies, where tumor blood supply is often associated with disease aggressiveness and prognosis [2].

Imaging has become an indispensable tool in evaluating breast vascularity. While traditional imaging methods like mammography and ultrasound provide valuable information about breast structure, they fail to reveal the intricate details of vascular networks [3]. Advanced imaging techniques such as magnetic resonance imaging (MRI), especially when combined with contrast agents, have revolutionized this field. Dynamic contrast-enhanced MRI (DCE-MRI) offers unique insights into the vascular characteristics of breast tissue, allowing for a more detailed analysis of blood flow, vessel permeability, and the presence of angiogenesis [4]. It captures real-time changes in contrast agent distribution, providing valuable functional information that aids in distinguishing benign from malignant lesions and assessing treatment response in breast cancer patients [4].

This review aims to explore the role of DCE-MRI in enhancing our understanding of breast vascularity. It will cover the principles underlying DCE-MRI, its application in both normal and diseased breast tissue, and how it contributes to improving the diagnosis and management of breast cancer. Additionally, we will discuss the quantitative analysis of vascularity, the emerging applications of DCE-MRI in breast imaging, and the limitations of this technique. Through this comprehensive review, we aim to highlight the importance of DCE-MRI in breast vascularity assessment and its potential to shape future breast cancer diagnostics and treatment strategies.

## Review

Anatomy and physiology of breast vascularity

The breast primarily receives its arterial blood supply from branches of the internal thoracic (mammary) artery, intercostal arteries, and the lateral thoracic artery. These arteries branch extensively within the breast, sending perforating branches deep into the parenchyma. Additionally, the superior breast parenchyma is supplied by branches of the subclavian and axillary arteries [5]. The venous anatomy mirrors the arterial structure, with paired and venous branches. Superficial veins drain the breast's center and periphery, forming a venous plexus known as the circulus venosus of Haller. Typically, breast veins lack valves, and intramammary venous anastomoses are common. During puberty, breast development is marked by increased blood flow and vascular density, which supports the growth of mammary glands [6]. In pregnancy and lactation, the blood supply to the breast significantly increases to accommodate the growing mammary glands and the demands of lactation. After menopause, breast vascularity generally decreases compared to premenopausal levels. In breast cancer, tumors induce angiogenesis, leading to higher vascular density and altered vascular morphology [7]. Increased microvessel density is linked to more aggressive cancer biology and poorer prognosis. DCE-MRI can evaluate these vascular changes, with features such as prominent ipsilateral vascularity and marked background parenchymal enhancement (BPE) serving as predictors for early and late recurrence, respectively [7].

Principles of DCE-MRI

**Figure 1:**
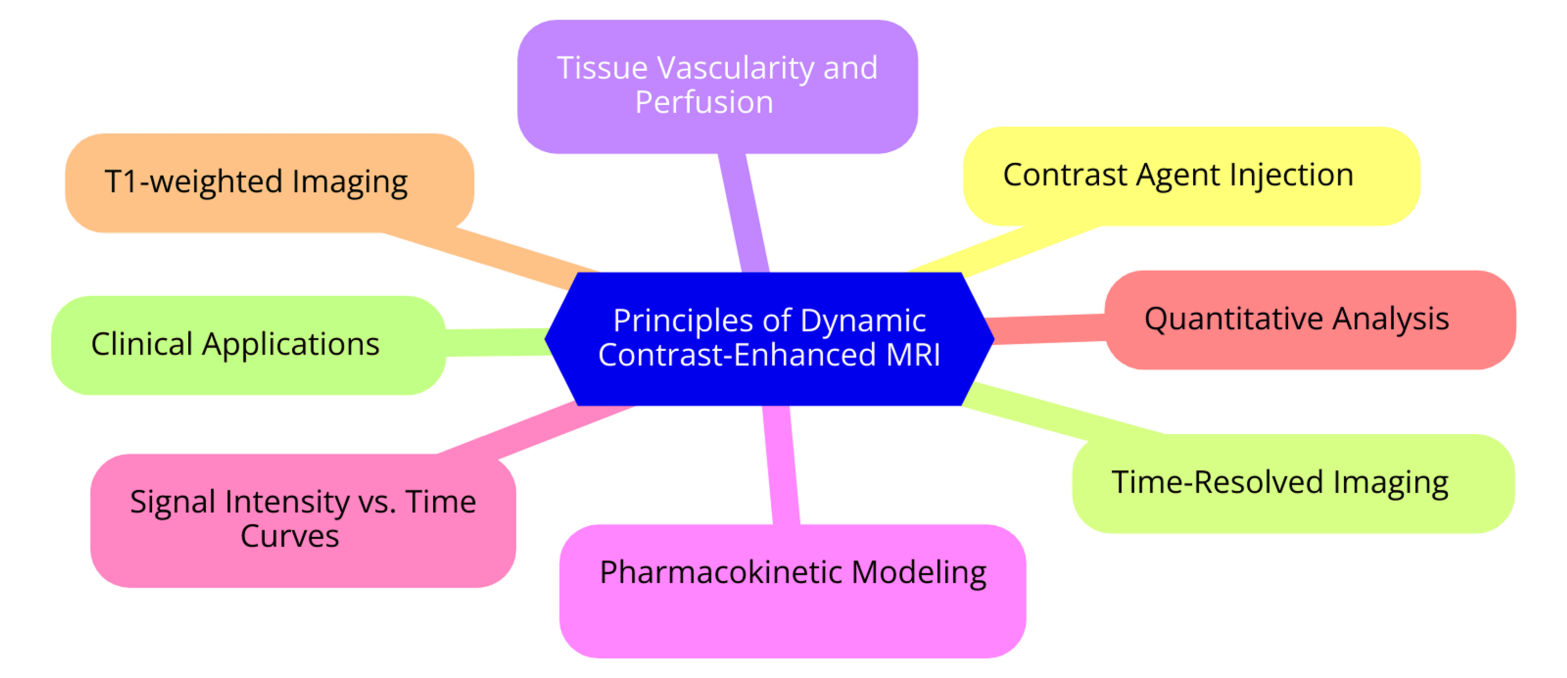
The principles of dynamic contrast-enhanced MRI

Dynamic DCE-MRI is a highly effective imaging technique used to evaluate breast vascularity, especially in the context of breast cancer. This overview outlines the fundamental principles of DCE-MRI, including the underlying technology, its role in capturing vascular patterns, the contrast agents used, and the essential timing and acquisition protocols for dynamic imaging [8]. MRI employs strong magnetic fields and radio waves to produce detailed images of internal organs and tissues. DCE-MRI builds on this by introducing a paramagnetic contrast agent that modifies the magnetic properties of nearby water molecules in tissues [9]. This modification results in changes in the MR signal intensity, which enables visualization of blood flow and vascular structures. The process involves obtaining images before and after administering the contrast agent. As the agent circulates and infiltrates the tissue, it enhances the signal, allowing for an assessment of tissue perfusion and vascularity. The variations in signal intensity over time are analyzed to derive kinetic parameters that reflect physiological characteristics such as blood flow and vessel permeability [10]. DCE-MRI assesses breast vascular patterns by analyzing the temporal dynamics of contrast agent enhancement in breast tissues. After injecting the contrast agent, imaging sequences capture the agent's initial uptake and subsequent washout [11]. The enhancement patterns are classified into kinetic curves: Type I curves indicate a progressive increase, in contrast, uptake, typically associated with benign lesions; Type II curves show initial uptake followed by a plateau, suggesting indeterminate lesions; and Type III curves reveal rapid uptake and washout, which are strongly correlated with malignant lesions. These curves provide valuable insights into vascularity and tumor biology, which are crucial for accurate diagnosis and treatment planning [11]. The contrast agents most commonly used in DCE-MRI are gadolinium-based compounds selected for their paramagnetic properties that enhance the MRI signal. Gadolinium-diethylenetriaminepentaacetic acid (DTPA) is frequently used because it provides clear images of vascular structures and tumor perfusion. Ongoing research is focused on developing newer formulations and agents to improve specificity and minimize potential side effects associated with gadolinium, thereby ensuring safer and more effective imaging [10]. DCE-MRI protocols typically have three phases: pre-contrast, early, and delayed post-contrast imaging. The pre-contrast phase establishes baseline images for comparison. The early post-contrast phase involves high temporal resolution imaging (four to eight seconds per image) to capture the initial contrast agent uptake, which is crucial for assessing vascular dynamics. The delayed post-contrast phase includes images taken at intervals after the initial uptake to observe the contrast agent's washout, offering further information on tissue characteristics [12]. Total scan times can range from 60 to 120 seconds per phase, depending on the specific protocol and desired spatial resolution. Advanced techniques, such as ultrafast DCE-MRI, utilize accelerated imaging methods to enhance temporal resolution while maintaining spatial clarity, providing a more detailed analysis of vascular patterns in breast tissues [13]. The principles of dynamic contrast-enhanced MRI are illustrated in Figure 1.

DCE-MRI in normal breast tissue

Dynamic DCE-MRI is essential for evaluating normal breast tissue, particularly in understanding enhancement patterns, hormonal changes' effects, and vascularity variations throughout the menstrual cycle. One key phenomenon in healthy breast tissue is background parenchymal enhancement (BPE) [8]. BPE refers to the enhancement of normal breast tissue after administering a contrast agent during DCE-MRI. This enhancement is categorized into four qualitative breast imaging reporting and data system (BI-RADS) categories: minimal, mild, moderate, and marked. The degree of BPE reflects the physiological activity of the breast tissue and is influenced by factors such as tissue vascularity, permeability, and hormonal levels [14]. Enhancement kinetics in normal breast parenchyma can be quantified through various measures, including the wash-in slope and signal enhancement ratio. These provide insights into the tissue’s vascular characteristics and may be associated with breast cancer risk by indicating underlying physiological changes [15]. Hormonal fluctuations, particularly those related to the menstrual cycle, significantly impact breast vascularity and the enhancement patterns observed in DCE-MRI. Estrogen and progesterone levels vary throughout the menstrual cycle, affecting breast tissue density and vascularity [16]. For instance, increased vascularity and glandular proliferation can lead to heightened BPE during the luteal phase, which may complicate MRI interpretation in some patients. Radiologists and clinicians must understand these hormonal influences when assessing DCE-MRI results, as they help differentiate between normal physiological changes and potential pathological conditions [17]. Research has shown that DCE-MRI enhancement characteristics can vary significantly throughout the menstrual cycle. Lower hormone levels may correspond to reduced vascularity and minimal BPE in the follicular phase. In contrast, heightened hormone levels during the luteal phase can increase vascularity and more pronounced BPE. This cyclical variation underscores the importance of carefully timing MRI examinations about the menstrual cycle to prevent misinterpretation of normal physiological changes as pathological conditions. By accounting for these factors, healthcare providers can enhance the accuracy of breast imaging interpretations and improve patient management [18]. Key parameters and findings of DCE-MRI in normal breast tissue are summarized in Table 1.

**Table 1:** Key parameters and findings of DCE-MRI in normal breast tissue

Parameter	Description	Findings in Normal Breast Tissue
Breast Composition [19]	The percentage of fibroglandular tissue and fat content in the breast.	Normal breast tissue shows a high fat content and lower fibro glandular density.
Vascular Enhancement [16]	The degree of contrast agent uptake in breast vessels.	Minimal to moderate enhancement with gradual contrast washout.
Enhancement Pattern [16]	The temporal changes in signal intensity after contrast administration.	Homogeneous or gradual enhancement in glandular tissue.
Kinetics [20]	Time-related behavior of contrast uptake and washout in the tissue.	Slow initial uptake with gradual washout, indicative of non-malignant tissues.
Type of Enhancement Curve [16]	Classification of signal intensity curve post-contrast (Type I, II, III).	Type I (persistent) is common in normal breast tissue, reflecting a benign pattern.
Background Parenchymal Enhancement (BPE) [21]	The enhancement of normal fibroglandular tissue after contrast injection.	Mild to moderate BPE is typical in premenopausal women; low to minimal BPE in postmenopausal women.
Hormonal Influence [17]	Impact of hormonal fluctuations (menstrual cycle, hormone replacement therapy) on breast tissue.	Variations in enhancement due to hormonal changes, with increased BPE during the luteal phase.
Fibroglandular Tissue Density [22]	Relative density of fibroglandular tissue on imaging.	Lower in postmenopausal women and higher in younger premenopausal women.
Symmetry [23]	Comparison of enhancement between both breasts.	Symmetric enhancement in normal tissue; asymmetry may warrant further evaluation.
Artifacts [24]	Image distortions or anomalies unrelated to pathology.	Common artifacts include motion artifacts and phase-encoding artifacts, typically corrected.

DCE-MRI in benign breast conditions

DCE-MRI is pivotal in evaluating benign breast conditions, particularly in distinguishing these from malignant lesions. Several benign conditions exhibit altered vascularity that can be effectively assessed using DCE-MRI. Notable examples include fibroadenomas, cysts, and pseudoangiomatous stromal hyperplasia (PASH) [8]. Fibroadenomas, being the most common benign breast tumors, are characterized by increased vascularity. On DCE-MRI, they typically display a type I kinetic curve, which indicates a slow and steady enhancement pattern without significant washout. Conversely, simple cysts generally show minimal enhancement and lack significant vascularity on DCE-MRI, though complex cysts may present some vascularity, complicating their assessment [25]. DCE-MRI provides distinct enhancement patterns that aid in characterizing benign breast lesions.

Most benign lesions, such as fibroadenomas, exhibit a type I kinetic curve with a gradual increase in signal intensity and minimal washout. Some benign lesions may show a plateau phase following initial enhancement, indicating a more complex vascular pattern and classifying them as type II kinetic curves [26]. Although type III curves are predominantly associated with malignant lesions, they can occasionally be observed in benign conditions, necessitating careful interpretation. Research indicates that a significant majority of benign lesions about 94.4 % demonstrate type I curves, underscoring the reliability of DCE-MRI in differentiating benign conditions from malignant ones based on enhancement kinetics [27]. Differentiating benign vascularity from malignancy using DCE-MRI involves several key factors. Kinetic analysis is crucial, as the shape of the time-signal intensity curve plays a vital role in diagnosis. Benign lesions typically exhibit stable enhancement patterns (type I), while malignant lesions often show rapid initial enhancement followed by washout (type III). Additionally, the degree of enhancement is informative; malignant lesions generally display higher maximum relative enhancement and a more significant washout rate than benign lesions, which usually have lower washout rates and less pronounced enhancement [28]. Integrating DCE-MRI with other imaging techniques, such as diffusion-weighted imaging (DWI) and apparent diffusion coefficient (ADC) mapping, enhances diagnostic accuracy. Malignant lesions typically present with lower ADC values due to higher cellularity, whereas benign lesions show higher ADC values, reflecting less restricted diffusion [29]. Overall, DCE-MRI is a valuable tool for assessing benign breast conditions, offering critical insights into vascularity and enhancement patterns that aid in differentiating benign from malignant lesions. Careful analysis of kinetic curves and enhancement characteristics is essential for accurate diagnosis and effective management of breast lesions [30]. Key parameters and findings of DCE-MRI in benign breast conditions are summarized in Table 2.

**Table 2:** Key Parameters and findings of DCE-MRI in benign breast conditions

Parameter	Description	Findings in Benign Breast Conditions
Lesion Morphology [16]	Shape, size, and margins of the lesion seen on imaging.	Typically round or oval with well-defined, smooth margins.
Vascular Enhancement [31]	Degree of contrast agent uptake in the lesion.	Mild to moderate enhancement; less aggressive compared to malignant lesions.
Enhancement Pattern [23]	Temporal changes in signal intensity post-contrast.	Homogeneous or heterogeneous enhancement is usually not as intense as in malignancies.
Kinetic Curve Patterns [32]	Time-intensity curves describing contrast uptake and washout.	Type I (persistent) or Type II (plateau) curves are common, indicating a benign nature.
Background Parenchymal Enhancement (BPE) [33]	Enhancement of the normal fibroglandular tissue after contrast injection.	Mild to moderate BPE may be influenced by hormonal status.
Fibroadenoma [16]	Common benign breast tumor.	Shows slow, persistent enhancement with well-defined borders and a Type I or II kinetic curve.
Cyst [34]	Fluid-filled benign lesion.	Appears as non-enhancing, circumscribed, or with minimal peripheral enhancement.
Fibrocystic Changes [35]	A benign condition involving fibrous and cystic tissue in the breast.	Mild diffuse enhancement, with areas of cystic non-enhancement.
Intraductal Papilloma [36]	Benign growth within a milk duct.	It may show moderate enhancement with a central duct pattern and smooth, well-defined margins.
Sclerosing Adenosis [37]	Benign condition with excessive growth of tissues in the breast lobules.	Enhancing lesions with a spiculated or irregular shape may mimic malignancy but show benign kinetic curves.
Hormonal Influence [38]	Impact of hormonal changes on benign breast lesions.	Hormonal fluctuations may affect BPE and enhancement patterns, especially in premenopausal women.
Artifacts [39]	Image distortions unrelated to pathology.	Motion or phase-encoding artifacts may be present and require correction or rescan.
Symmetry [16]	Comparison of enhancement and lesion characteristics between both breasts.	Symmetry in benign conditions; significant asymmetry may require further evaluation.

DCE-MRI in breast cancer

DCE-MRI has become a vital tool in evaluating breast vascularity, particularly in the context of breast cancer diagnosis and treatment. Angiogenesis, the formation of new blood vessels, is a crucial process in the initiation and progression of invasive breast cancer [40]. High microvessel density (MVD) is a significant prognostic marker, indicating aggressive cancer behavior and poor survival rates in patients with invasive breast cancers. Increased vascular blood flow supports tumor growth and facilitates the hematogenous spread of malignant cells [40]. DCE-MRI is employed to assess various aspects of breast cancer biology, including vascular density, vascular morphology, and the aggressiveness of the disease. Tumors with elevated MVD exhibit more rapid contrast clearance, as indicated by peak signal enhancement ratio (SER) and washout fraction (WF) on DCE-MRI [41]. Prominent increased ipsilateral whole-breast vascularity is linked to early cancer recurrence and a worse prognosis. DCE-MRI provides a non-invasive means to evaluate breast cancer angiogenesis, which can help stratify tumor biology and optimize treatment strategies. Key kinetic features such as initial peak percent enhancement (PE), SER, functional tumor volume (FTV), and WF, along with radiomic features reflecting tumor morphology, signal intensity, and texture, correlate with MVD as measured by histologic CD31 immunostaining [41]. The types of kinetic curves and enhancement patterns observed on DCE-MRI, such as the initial enhancement pattern and background parenchymal enhancement (BPE), can predict breast cancer recurrence. Moderate or marked BPE is notably predictive of late and overall recurrence. DCE-MRI has proven highly sensitive and transformative for the early detection of breast cancer, particularly in women at increased risk [42]. Advances in ultrafast DCE-MRI techniques provide high temporal resolution, capturing rapid changes in vascularity shortly after contrast administration. This enhanced resolution improves diagnostic accuracy and offers valuable prognostic information regarding tumor characteristics and treatment response [42]. Key parameters and findings of DCE-MRI in breast cancer are summarized in Table 3.

**Table 3:** Key parameters and findings of DCE-MRI in breast cancer

Parameter	Description	Findings in Breast Cancer
Lesion Morphology [43]	Shape, size, and margins of the lesion seen on imaging.	Irregular or spiculated shape with poorly defined, irregular margins.
Vascular Enhancement [44]	Degree of contrast agent uptake in the lesion.	Rapid, intense enhancement due to increased angiogenesis; typically, more aggressive than benign lesions.
Enhancement Pattern [45]	Temporal changes in signal intensity post-contrast.	Heterogeneous enhancement, with necrosis or central non-enhancement areas, is often present.
Kinetic Curve Patterns [23]	Time-intensity curves describing contrast uptake and washout.	Type III (washout) curve common, indicating malignancy with rapid uptake and fast contrast washout.
Background Parenchymal Enhancement (BPE) [46]	Enhancement of the normal fibroglandular tissue after contrast injection.	BPE may vary; increased BPE can sometimes obscure small lesions.
Tumor Invasion [47]	Spread of the tumor into adjacent tissues.	It may show invasion into the skin, pectoral muscles, or chest wall; a spiculated appearance suggests invasive cancer.
Lymph Node Involvement [48]	Spread of cancer to axillary or other regional lymph nodes.	Enlarged, enhancing lymph nodes with irregular borders may indicate metastatic involvement.
Tumor Necrosis [49]	Presence of necrotic tissue within the tumor.	Central areas of non-enhancement in the tumor mass indicate necrosis.
Multifocal/Multicentric Disease [50]	Presence of multiple tumors in one or more quadrants of the breast.	Multiple areas of enhancement in different quadrants (multicentric) or the same quadrant (multifocal).
Hormonal Influence [51]	Impact of hormonal status on imaging.	It may show variation in BPE, but malignancy typically retains aggressive enhancement patterns regardless.
Tumor Size and Volume [52]	Size and volume of the primary tumor.	Larger tumors generally show more intense enhancement and may involve surrounding structures.
Symmetry [53]	Comparison of enhancement and lesion characteristics between both breasts.	Asymmetric enhancement in the affected breast, often with additional satellite lesions.
Artifacts [54]	Image distortions unrelated to pathology.	Potential artifacts (motion, phase-encoding) can be minimized to avoid misinterpretation of malignant findings.

DCE-MRI for monitoring treatment response

DCE-MRI has become a crucial tool for monitoring treatment response in breast cancer patients undergoing various therapies. This imaging modality enables non-invasive assessment of tumor vascularity, closely associated with tumor angiogenesis and growth. By evaluating changes in DCE-MRI parameters during and after treatment, clinicians can gain insights into the effectiveness of the administered therapy and make informed decisions regarding patient management [55]. DCE-MRI is used to monitor alterations in tumor vascularity during chemotherapy, radiation therapy, and targeted therapies. For instance, during chemotherapy, DCE-MRI can track the effects of neoadjuvant chemotherapy on tumor vascularity. Tumors that respond favorably to chemotherapy often transition from a heterogeneous to a more homogeneous internal composition, accompanied by a decrease in peak enhancement post-treatment [56]. Similarly, during radiation therapy, DCE-MRI assesses changes in tumor vascularity, with studies revealing decreases in transfer constant and extracellular volume fraction, reflecting significant antiangiogenic effects. Additionally, DCE-MRI evaluates the impact of targeted therapies, such as bevacizumab, where reductions in pharmacokinetic parameters like the inflow transfer rate constant and extracellular volume fraction have been observed [57]. Beyond monitoring treatment response, DCE-MRI parameters can also predict treatment outcomes based on vascular changes. For example, a novel morpho-physiological tumor score (MPTS) derived from pre-treatment DCE-MRI images has demonstrated the ability to predict responses to neoadjuvant chemotherapy and hormone therapy in patients with locally advanced breast cancer [58]. DCE-MRI can also differentiate between trastuzumab-responsive and trastuzumab-resistant human epidermal growth factor receptor 2 (HER2)-overexpressing breast cancer xenografts, providing valuable insights into mechanisms of treatment resistance [58]. Additionally, DCE-MRI plays a vital role in assessing the risk of cancer recurrence. Specific DCE-MRI features, such as prominent ipsilateral vascularity and particular kinetic curve types, have been linked to the timing of recurrence. By identifying high-risk patients based on DCE-MRI findings, clinicians can tailor follow-up strategies and implement appropriate interventions to mitigate recurrence risk [59].

Quantitative analysis of breast vascularity using DCE-MRI

DCE-MRI has become a crucial tool for the quantitative analysis of breast vascularity, particularly in breast cancer diagnosis and treatment. This advanced imaging technique allows for the detailed assessment of various perfusion metrics and kinetic modeling parameters, essential for understanding tumor biology and guiding clinical decisions [8]. A key technique in DCE-MRI is the calculation of the volume transfer constant (Ktrans), which measures the rate at which the contrast agent moves from the blood plasma into the extracellular extravascular space. Elevated Ktrans values are often seen in malignant tumors, reflecting increased vascular permeability due to angiogenesis [41]. Another important parameter is the extracellular-extravascular volume (Ve), which indicates the extracellular space volume relative to the tissue volume. We provide insights into the tumor microenvironment and can reveal the presence of necrosis or edema. Blood flow (F) is also assessed to understand how quickly blood circulates through the tumor vasculature. This is critical for evaluating the delivery of therapeutic agents and oxygen, ultimately impacting treatment efficacy [41]. In addition to Ktrans, Ve, and blood flow, several other kinetic parameters are used to quantify breast vascularity. The initial peak percent enhancement (PE) measures the initial uptake of the contrast agent, reflecting the tumor’s vascularity. The signal enhancement ratio (SER) compares the signal intensity of the tumor with that of the surrounding tissue, providing insights into relative vascularity. The washout fraction (WF) quantifies the rate at which the contrast agent is cleared from the tumor, with faster washout often associated with more aggressive tumor behavior [60]. Integrating artificial intelligence (AI) and machine learning (ML) into DCE-MRI analysis is transforming the field of breast vascularity quantification. AI technologies enable automated feature extraction, allowing algorithms to identify and quantify relevant imaging features from DCE-MRI data. This automation reduces human error and increases measurement reproducibility [61]. Radiomics, which involves extracting many quantitative features from medical images, benefits from AI advancements. Radiomic features derived from DCE-MRI have been shown to correlate with histopathological findings such as microvessel density (MVD), offering valuable insights into tumor angiogenesis and aggressiveness. Furthermore, advanced machine learning models enhance the analysis of complex DCE-MRI data, improving the differentiation between benign and malignant lesions based on vascular characteristics [61].

Emerging applications of DCE-MRI in breast vascularity

DCE-MRI is increasingly recognized for its valuable applications in breast cancer management, particularly in personalized treatment, high-risk screening, and integration with other imaging modalities [62]. This advanced imaging technique plays a crucial role in tailoring individualized treatment regimens for breast cancer patients. DCE-MRI enables the early prediction of treatment responses, especially to neoadjuvant chemotherapy (NAC). Research has shown that quantitative pharmacokinetic parameters derived from DCE-MRI can effectively predict the residual cancer burden following treatment. This capability allows clinicians to make informed therapeutic decisions, optimizing management strategies based on the specific characteristics of each patient's tumor [62]. In addition to personalizing treatment, DCE-MRI is highly effective in detecting invasive breast cancer, making it an essential tool for high-risk screening and early detection. Its ability to visualize the vascularity and permeability of lesions enhances diagnostic accuracy, particularly in women with dense breast tissue where traditional mammography may be less effective. By incorporating DCE-MRI into screening protocols, healthcare providers can achieve earlier identification of malignancies, which is crucial for timely intervention and improved patient outcomes [63]. Furthermore, combining DCE-MRI with other imaging techniques, such as diffusion-weighted imaging (DWI) and apparent diffusion coefficient (ADC) mapping, significantly enhances diagnostic capabilities for breast lesions. While DCE-MRI provides insights into vascularity and perfusion, DWI offers valuable information on cellular density and the tissue microenvironment. This multiparametric approach allows for a more comprehensive evaluation of breast tumors, improving the differentiation between benign and malignant lesions and enhancing diagnostic specificity [64]. Emerging applications of DCE-MRI in breast vascularity are summarized in Table 4.

**Table 4:** Emerging applications of DCE-MRI in breast vascularity

Application	Description	Potential Impact on Breast Vascularity Assessment
Tumor Angiogenesis Evaluation [65]	Assessing the formation of new blood vessels within tumors using contrast-enhanced imaging.	Improved detection and characterization of angiogenesis in early breast cancer, allowing for better prognosis.
Functional Imaging Biomarkers [66]	Using imaging to develop biomarkers that reflect tumor biology and vascular characteristics.	Provides quantitative data on vascular permeability and blood flow, enhancing precision in treatment planning.
Predicting Tumor Response to Therapy [67]	Utilizing DCE-MRI to assess changes in tumor vascularity before and after neoadjuvant therapies.	Early prediction of treatment effectiveness by observing changes in vascular dynamics, optimizing patient management.
Vascular Heterogeneity Mapping [68]	Identifying spatial variations in blood supply within tumors to understand intratumoral heterogeneity.	Aids in targeting the most aggressive regions of the tumor, potentially improving surgical or radiotherapy outcomes.
Personalized Treatment Planning [69]	Using DCE-MRI to tailor treatment based on individual vascular characteristics of tumors.	Enables more personalized approaches by mapping tumor blood supply, leading to more effective and targeted therapies.
Monitoring Anti-Angiogenic Therapies [70]	Evaluating the efficacy of drugs that inhibit blood vessel growth in tumors by tracking vascular changes.	Non-invasive monitoring of the success of anti-angiogenic agents in real-time, helping to adjust treatment strategies.
Assessment of Microvascular Density [71]	Quantifying microvascular density in breast tissue to assess the aggressiveness of breast lesions.	Offers insights into the biological behavior of tumors, aiding in distinguishing between benign and malignant lesions.
Vascularity in High-Risk Populations [72]	Exploring vascular patterns in individuals with genetic predispositions (BRCA mutations, etc.).	Provides early detection of abnormal vascularity in high-risk patients, enabling preventive interventions.
Differentiating Tumor Subtypes [73]	Using DCE-MRI to differentiate between molecular subtypes of breast cancer based on vascular patterns.	Assists in identifying more aggressive subtypes like triple-negative breast cancer through distinct vascular signatures.
Breast Density and Vascularity Correlation [74]	Investigating the relationship between breast density and vascular patterns in cancer development.	Enhances understanding of how breast density influences vascularity, potentially improving risk stratification methods.

Limitations and challenges of DCE-MRI

DCE-MRI is a powerful tool for assessing breast vascularity, particularly in the context of breast cancer. However, it does have limitations and challenges that must be considered. One significant technical issue is the presence of motion artefacts. DCE-MRI studies, which often extend over several minutes, are susceptible to patient movement and physiological factors such as rectal peristalsis and bladder filling. These can lead to misregistration between consecutive slices [8], introducing noise into the wash-in and washout curves and complicating the accurate fitting of pharmacokinetic models. Another challenge involves achieving optimal spatial and temporal resolution. High spatial resolution is necessary to avoid volume averaging and accurately image suspicious lesions, while high temporal resolution is crucial for capturing rapid changes in vascularity immediately after contrast administration. Balancing these requirements can increase scan time and reduce the signal-to-noise ratio, further complicating the imaging process [41]. Interpretation of DCE-MRI findings can also be challenging due to overlapping enhancement patterns. Increased vascular permeability can occur in various conditions, including prostatitis, highly vascularized benign prostatic hyperplasia (BPH) nodules, and malignant tumors [44]. This overlap can lead to false-positive results, complicating the diagnostic process. Additionally, the location of tumours can impact DCE-MRI results. For instance, anterior hypovascular transitional zone tumours may not show significant DCE-MRI uptake due to limited blood supply, potentially leading to false-negative findings. Post-biopsy changes, such as residual haemorrhage, can also result in false-positive and false-negative results, further complicating image interpretation [44]. Accessibility and cost-effectiveness are additional concerns. Adding DCE-MRI to standard imaging protocols increases overall costs due to the requirement for gadolinium-based contrast media [75]. There are also safety concerns related to the long-term deposition of gadolinium in the brain and kidneys, even in patients with normal renal function. Clinicians must carefully weigh the benefits and risks of using gadolinium-based contrast agents in breast imaging. Moreover, the availability of high-quality DCE-MRI, particularly with 3T scanners, varies across institutions, making it difficult to achieve consistent results across different centres [75].

Future directions

DCE-MRI has become a pivotal tool in assessing breast vascularity, particularly for breast cancer diagnosis and treatment. This review integrates insights from various studies on the role of DCE-MRI in evaluating breast vascularity and its impact on breast cancer management [76]. DCE-MRI is instrumental in assessing angiogenesis, a key factor in breast cancer progression. Angiogenesis, which involves the formation of new blood vessels, is closely associated with tumour growth and metastasis. High microvessel density (MVD) is a prognostic marker indicating aggressive cancer behaviour and poorer survival rates in patients with invasive breast cancers [77]. DCE-MRI provides detailed insights into vascular density and morphology, essential for understanding tumour biology. The technique allows for the non-invasive evaluation of breast cancer angiogenesis through various kinetic parameters, including initial peak per cent enhancement (PE), signal enhancement ratio (SER), and washout fraction (WF). Recent advancements in radiomics facilitate the extraction of quantitative features from DCE-MRI images, improving the assessment of vascular characteristics and tumour heterogeneity [40]. This computational analysis aids in the early detection of vascularization and enhances the understanding of tumour behaviour. Emerging techniques like ultrafast DCE-MRI offer a high temporal resolution, capturing rapid changes in vascularity shortly after contrast administration, which enhances diagnostic accuracy and provides prognostic information related to tumour characteristics and treatment response [40]. Integrating DCE-MRI into clinical practice has significant implications. By correlating DCE-MRI features with MVD and other histopathological markers, clinicians can better stratify patients for personalized treatment approaches, thereby optimizing therapeutic outcomes [40]. Certain DCE-MRI features, such as prominent ipsilateral vascularity and specific kinetic curve types, have been linked to the timing of cancer recurrence, which is crucial for patient management and follow-up. Additionally, combining DCE-MRI with other imaging modalities, such as apparent diffusion coefficient (ADC) mapping, enhances diagnostic efficacy in differentiating benign from malignant breast lesions, aiding early detection and intervention [40]. Several promising future directions could further enhance the understanding and clinical applications of breast DCE-MRI. Developing techniques for higher spatial resolution could improve the visualization of small vessels and enable a more detailed assessment of tumour vascularity [78]. Utilizing larger molecular-weight contrast agents, less likely to leak than low-molecular-weight agents, may provide more specific information about tumour angiogenesis. Validating DCE-MRI techniques across diverse patient populations, including various ethnicities and age groups, can ensure the generalizability of findings and guide personalized treatment approaches. Furthermore, correlating DCE-MRI features with tumour biology and treatment outcomes could help identify imaging biomarkers that predict response to antiangiogenic and other targeted therapies [78]. Addressing these future directions will advance the role of DCE-MRI in breast cancer management, leading to improved patient outcomes through personalized treatment strategies and more effective monitoring of therapeutic response [79].

## Conclusions

In conclusion, understanding breast vascularity is crucial in diagnosing and managing breast diseases, particularly breast cancer, where vascular changes often indicate tumor aggressiveness and treatment response. DCE-MRI has emerged as a powerful tool, offering detailed insights into breast tissue's vascular architecture and perfusion patterns. Its ability to distinguish between benign and malignant lesions, monitor therapeutic outcomes, and provide quantitative data on vascular properties makes it invaluable in clinical practice. Despite certain limitations, such as accessibility and cost, DCE-MRI holds significant promise for enhancing breast cancer diagnosis, improving personalized treatment approaches, and advancing research into breast vascularity. As technological advancements continue, the role of DCE-MRI in breast imaging is poised to expand, offering even greater precision in assessing vascular changes and contributing to better patient outcomes.
